# Sudden Death in Adults: A Practical Flow Chart for Pathologist Guidance

**DOI:** 10.3390/healthcare9070870

**Published:** 2021-07-09

**Authors:** Francesco Sessa, Massimiliano Esposito, Giovanni Messina, Giulio Di Mizio, Nunzio Di Nunno, Monica Salerno

**Affiliations:** 1 Department of Clinical and Experimental Medicine, University of Foggia, 71122 Foggia, Italy; giovanni.messina@unifg.it; 2 Department of Medical, Surgical and Advanced Technologies “G.F. Ingrassia”, University of Catania, 95121 Catania, Italy; massimiliano.esposito91@gmail.com (M.E.); monica.salerno@unict.it (M.S.); 3 Forensic Medicine, Department of Law, Economy and Sociology, Campus “S. Venuta”, Magna Graecia University, 88100 Catanzaro, Italy; giulio.dimizio@unicz.it; 4 Department of History, Society and Studies on Humanity, University of Salento, 73100 Lecce, Italy; nunzio.dinunno@icloud.com

**Keywords:** sudden death (SD), sudden cardiac death (SCD), autopsy, molecular autopsy, genetics, post-mortem investigation, practical flowchart in SD

## Abstract

The medico-legal term “sudden death (SD)” refers to those deaths that are not preceded by significant symptoms. SD in apparently healthy individuals (newborn through to adults) represents a challenge for medical examiners, law enforcement officers, and society as a whole. This review aims to introduce a useful flowchart that should be applied in all cases of SD. Particularly, this flowchart mixes the data obtained through an up-to-date literature review and a revision of the latest version of guidelines for autopsy investigation of sudden cardiac death (SCD) in order to support medico-legal investigation. In light of this review, following the suggested flowchart step-by-step, the forensic pathologist will be able to apply all the indications of the scientific community to real cases. Moreover, it will be possible to answer all questions relative to SD, such as: death may be attributable to cardiac disease or to other causes, the nature of the cardiac disease (defining whether the mechanism was arrhythmic or mechanical), whether the condition causing SD may be inherited (with subsequent genetic counseling), the assumption of toxic or illicit drugs, traumas, and other unnatural causes.

## 1. Introduction

The medico-legal term “sudden death (SD)” (also called “sudden and unexpected natural death”) refers to those deaths that are not preceded by significant symptoms. The term thus used obviously excludes violent or traumatic deaths. There is no universally accepted definition of sudden death, and time periods ranging from 1 to 48 h have been used in several countries. For example, the World Health Organization (WHO) definition of SD is a “death occurring within 24 h after the onset of the symptoms” [1]; while the Association for European Cardiovascular Pathology has defined SD as “a natural death that occurs within 6 h of the beginning of symptoms in an apparently healthy subject or in one whose disease is not so severe that a fatal outcome would be expected” [2].

SD in apparently healthy individuals (from newborn through to adults) represents a very challenging event for medical examiners, law enforcement officers, and society as a whole [3]. It is particularly difficult to interpret epidemiological data, considering the lack of standardization in death certificate coding and the variability in the definition of SD. Some causes of SD are identifiable through collecting several important pieces of evidence during the external examination, crime scene investigation, and autopsy [4,5,6]. Moreover, anamnesis and clinical data should be collected in order to identify the exact cause of death [7,8]. Nevertheless, in many cases of SD that present to the medical examiner or coroner, all collected data do not reveal the cause of death [9].

Several SDs are not necessarily “unexpected”, and some unexpected deaths are not necessarily “sudden”: for this reason, it is extremely important that these autopsies be carried out, and that they are conducted properly. Notably, autopsy findings may have profound effects on the lives and welfare of the family of the deceased, law enforcement agencies, hospital authorities, and private corporations, including insurance companies [10].

In this scenario, this narrative review introduces a useful flowchart that should be applied in all cases of SD. In particular, this flowchart was obtained combining the data obtained through an up-to-date literature review and a revision of the latest version of guidelines for autopsy investigation of sudden cardiac death (SCD) [11] in order to support medico-legal investigations. The choice of the articles for this narrative review was made after evaluation by the authors following a screening of abstracts in the PUBMED database, using the following search terms: “Causes of SD”, “SD and Cardiovascular system”, “SD and Respiratory System”, “SD and Central Nervous System”, “SD and Abdominal causes”, “SD and Endocrine System”, “SD and Iatrogenic”, and “SD and Miscellaneous”.

## 2. Causes of Sudden Death

The most prevalent cause of death in the case of SD is related to cardiovascular diseases; nevertheless, when a subject suddenly dies, and the pathologist after the post-mortem examination is not able to identify abnormalities of the cardiac anatomy, a variety of conduction abnormalities without morphological evidence visible at autopsy may be suspected. In light of these considerations, other organs may be involved: these cases of SDs are usually defined as non-cardiac sudden death (nc-SD).

**Figure 1:**
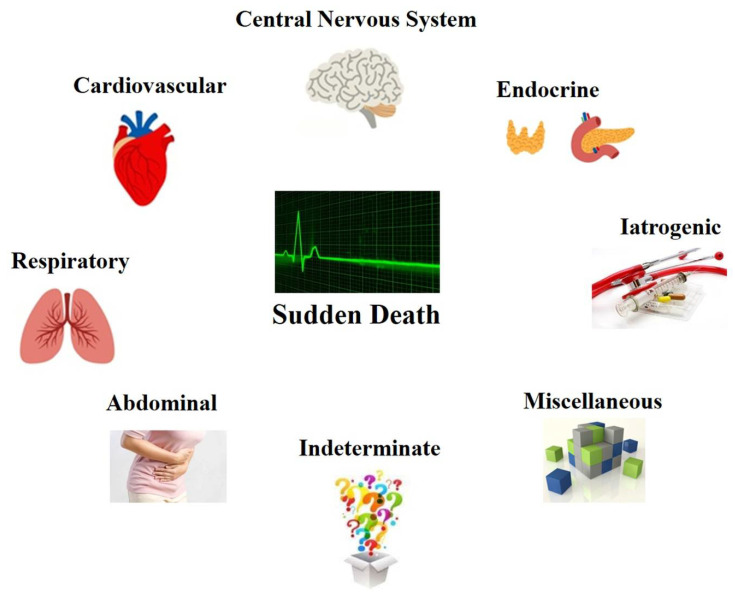
A classification of the possible cause of death in cases of SD.

Based on these considerations, SD may be classified under the criteria of the anatomical system involved. It is clear that following these criteria, some degree of overlapping is inevitable. In Figure 1, a system of classification of SD is proposed.

### 2.1. Cardiovascular System

The first cause of SD worldwide is cardiovascular diseases, accounting for approximately 90% of such cases [9]. These data are referred to developed countries such as the USA, Japan, and various European countries. When the cardiovascular system is involved, SCD is used. The definitions of SCD are not the same across the scientific community [12]. The incidence of SCD increases with age, varying from about 1 per 1000 per year in subjects of 35–40 years, 2 per 1000 per year by 60 years, and 200 per 1000 per year in the elderly [13,14]. The use of post-mortem imaging is very important in the classification of SCD. Based on these criteria, they may be divided into coronary and non-coronary causes:

- coronary artery diseases (CAD) represent the majority of cardiovascular deaths, even if the data about the percentage are various, ranging from 56.87% [15] to 80% [9]. The percentage of CAD deaths is closely related to age: indeed, it was highest in subjects over 40 years old. CAD may be further divided into atherosclerotic and non-atherosclerotic types. In this subdivision, the atherosclerotic type accounts for most cases, while non-atherosclerotic coronary artery diseases are included in congenital abnormalities, embolism, arteritis, dissecting aneurysms, and external compression or ostial obstruction;
- non-coronary cardiovascular diseases are strictly related to congenital anomalies, valvular heart diseases such as rheumatic heart disease and syphilis, hypertensive heart disease, myocarditis, ruptured aortic aneurysm (acute aortic dissection), and cardiomyopathy [16]. In a simplistic classification, SD due to cardiac genetic alterations could be subdivided into two main groups, channelopathies, and cardiomyopathies [17,18].

The so-called SCDs in the majority of cases are due to coronary artery disease. The post-mortem findings both at the gross examination and at the histological investigations frequently support this kind of diagnosis, describing a clinical picture of severe coronary artery atherosclerosis. They may be associated with coronary artery thrombosis, recent myocardial infarction, and myocardial fibrosis subsequent to different events such as infarction. Nevertheless, these findings are variable and relatively infrequent: for these reasons, they may not be considered decisive to validate the diagnosis [19].

The autopsy finding of critical coronary stenosis (defined as one or more of the major extramural coronary arteries with more than 75% narrowing of the luminal cross-section) is sufficient to invoke a diagnosis of SCD, and this is consistently detected in 90% or more of these patients. Death is believed to be due to rhythm disorders, i.e., dysrhythmias, in most of these cases. In the adult, it is estimated that 10 to 25% of SCD could be related to cardiac channelopathies [20].

Several risk factors for SCD have been reported [21], with age and sex representing two important factors: indeed, the risk of SD is greater in males and obviously increases with age. The death rate improves significantly in middle-old age, especially from age 45 to 64 years [22]. Another important factor is the presence of previous coronary artery disease [23]. Patients with known coronary artery disease had a fourfold greater incidence of SD. Nevertheless, about 55% of those dying suddenly had manifested no prior evidence of coronary artery disease. Another important heart disease closely related to SCD is left ventricular hypertrophy: as previously described, patients with ECG evidence of left ventricular hypertrophy had a 5-fold increased incidence of SD.

In this scenario, hypertension and blood cholesterol may be considered two important indirect risk factors in the insurgence of SCD events. Notably, men with systolic blood pressures >160 mm Hg have an incidence of SD three times greater than those who have systolic pressures <140 mm Hg; moreover, elevated cholesterol levels are generally regarded as a risk factor, even if, to date, no stepwise trend proportional to serum cholesterol has been noted [24]. Obviously, overweight and obese status is related to SCD. It has been described that the risk of this tragic event increases progressively with increased weight, arriving at more than doubled for those weighing 120% or more than their ideal weight [25]. Other important indirect factors that could be considered are cigarette smoking and alcohol/drug abuse. In particular, smokers had a 3-fold greater incidence of SD than non-smokers; moreover, the abuser (meaning smokers of >1 pack per day) had higher rates than did smokers of <1 pack per day [26].

In this context, an infrequent but always tragic event occurs when SD happens in an apparently healthy young adult from spontaneous causes. Although the causes of SD in young subjects are scarce, the most prevalent cause of death is related to cardiovascular disease, with primary arrhythmogenic disorders, atherosclerotic events, cardiomyopathies, and myocarditis as the main contributors. Indeed, in a recent article by Vos et al. [27], cardiovascular diseases and genetic arrhythmias accounted for about 50% of their SD cases. Indeed, based on international data, it is estimated that up to one-third of infantile and juvenile SCD may be explained by cardiac channelopathies [28,29,30,31]. The most frequent channelopathies include the long QT syndrome (LQTS), short QT syndrome (SQTS), catecholaminergic polymorphic ventricular tachycardia (CPVT), and Brugada syndrome (BrS) [32,33,34]. Moreover, in the case of SCD in young people, it could be possible to detect pathogenic mutations in genes encoding structural proteins. These mutations could determine several diseases such as hypertrophic cardiomyopathy, dilated cardiomyopathy, and arrhythmogenic cardiomyopathy (AC) [35]. The majority of these cardiomyopathies may be diagnosed at autopsy, considering that usually, anatomo-morphological changes in cardiac tissue are detected, especially in young adults [36]. Indeed, as recently described, a primary myocardial fibrosis at autopsy is strictly related to variants in genes associated with arrhythmogenic right ventricular cardiomyopathy, dilated cardiomyopathy, and hypertrophic cardiomyopathy; when autopsy does not show these findings, primary myocardial fibrosis may represent an alternative phenotypic expression of structural disease-associated genetic variants, or that risk-associated fibrosis was expressing before the primary disease [37]. Contrariwise, when this tragic event occurs in an infant, a structurally normal heart is usually reported in the post-mortem documentation [38].

One of the most difficult problems for forensic pathologists is the diagnose of SD in subjects with acute cardiac processes that progress rapidly, with non-specific symptoms, leading to death without evident morphological alterations. In these cases, innovative approaches are frequently proposed. For example, post-mortem magnetic resonance imaging could be one of the most promising tools to identify cardiac pathological alterations, highlighting evidence otherwise not visible with routine autopsy [39]. In the same way, the use of biochemical markers in cadaver fluids is frequently investigated as complementary indicators to help to reach valid conclusions about the cause of death. Although the ideal sampling site is debatable, several studies propose either pericardial fluid or peripheral veins as the location for the biological sample. A recent article suggests that cardiac troponin I (cTnI) values in pericardial fluid and the troponin ratio (pericardial fluid/serum ratio) may be helpful in SCD [40]. Finally, immunohistochemical investigation combined with western blot analysis is used to detect morphological changes in myocardial specimens of fatal SD, quantifying the effects of cardiac expression of inflammatory mediators (CD15, IL-1β, IL-6, TNF-α, IL-15, IL-8, MCP-1, ICAM-1, CD18, tryptase) and structural and functional cardiac proteins (troponin I and troponin C) [41].

When SD occurs in a young subject, it may occur during sports activities. In large post-mortem investigations studies of athlete populations in the United States, hypertrophic cardiomyopathy was the most common cardiovascular cause of SD [42]. The second most frequent cardiovascular cause of SD in the subjects practicing sports activities is congenital coronary-artery anomalies: in these cases, the artery originates from the wrong aortic sinus (more commonly, the left main coronary artery originates from Valsalva’s right sinus) [43]. Other causes of death are congenital cardiac malformations, such as congenital valvular disease, aortic stenosis, myxomatous mitral valve degeneration (typically associated with Marfan’s syndrome), as well as other causes such as myocarditis and coronary atherosclerotic disease [44,45,46].

A molecular autopsy should be considered fundamental in the case of SD in young people, seeking to always incorporate genetic testing into the post-mortem examination. Moreover, it has been proposed that community-based data aggregation and sharing should be mandatory, leading to an improved classification of genetic variants [47,48].

### 2.2. Respiratory System

The most important cause of death in the case of nc-SD involving the respiratory system is acute pulmonary embolism (APE). The symptoms of APE are various with a complex clinical picture. Indeed, APE is characterized by numerous clinical manifestations with a complex interaction between different organs. In this scenario, it is very difficult to make an immediate diagnosis [49].

Fatal pulmonary embolism (PE) represents the common cause of SD related to the respiratory system, usually resulting from a complication of deep venous thrombosis (DVT). Typical symptom and signs of PE is angina with pleuritic chest pain as a consequence of pleural involvement due to pulmonary infarction [50,51]. In fulminant PE, up to 90% of cardiac arrests occur within 1 to 2 h after the onset of symptoms [52]. The mortality rate related to PE is high and it is due to the principal causes: pulmonary mainstream obstruction and liberation of vasoconstrictive mediators from the thrombi [53]. For example, pulmonary thromboembolism has been identified as one of the common clinical pictures of COVID-19, justifying SD in several subjects who died from SARS-CoV-2 infection [54,55,56]. Other clinical pictures of APE are: symptoms similar to acute respiratory distress syndrome (ARDS) [57]; fever syndrome with or without pseudopneumonia [58]; acute right heart failure/shock/hypotension (often with epigastric pain) [59]; left heart failure (with pulmonary congestion) [60]; chest pain similar to pleuritic syndrome with or without hemoptysis (with or without effusion) [61]; similar to acute coronary syndrome (ACS) (with or without chest pain) [62]; syncope [63]; complete atrioventricular (AV) block with idioventricular rhythm [49]; persistent or paroxysmal atrial fibrillation (AF), atrial flutter, atrial tachycardia [64]; paroxysmal supraventricular tachycardia (PSVT) [49]; DVT and silent PE [65]; platypnea-orthodeoxia [66]; abdominal pain without acute abdomen [64]; and delirium [67].

Other causes of SD strictly related to the respiratory system are:

- Massive hemoptysis. The common causes of massive hemoptysis could be generated by tuberculosis, bronchiectasis, lung abscesses, and mycetomas [68]; another important cause of massive hemoptysis is lung cancer that could generate this symptoms in about 20% of patients [69]. Moreover, cystic fibrosis is reported to be another cause of massive hemoptysis [70].
- Severe pneumonia. This cause is considered one of the natural causes of nc-SD [71]. It could be generated both by viral and bacterial agents, generating different diseases involving myocardial ischemia, a maladaptive response to hypoxia, sepsis-related cardiomyopathy, or other phenomena [72,73]. It is interesting to note that the recent pandemic infection of SARS-CoV-2 could generate severe pneumonia with subsequent cardiac arrest [74].
- Asthma. In a recent study performed in Denmark in young people with uncontrolled asthma, Gullach et al. [75] described that in their cohort, the predominant cause of death was SCD followed by a fatal asthma attack. This clarified the concept that asthma could be considered as a trigger for underlying unknown heart diseases [76].
- Anaphylaxis. Refers to the event cascade that may follow exposure to a particular antigen and causing an acute multi-organ response, with cardiac, coronary/systemic arterial, and dermatological involvement [77]. Cardiovascular symptoms can be the sole manifestation of food allergies, especially in cases where the tragic event occurs during exercise [78]; in similar cases, death may mimic SCD and only a complete autopsy with a full histological and immunohistochemical investigation may disclose the exact cause of death.
- Airway obstruction. Another important cause of nc-SD is hypoxia secondary to pulmonary processes, including small and large airway obstruction (bronchospasm, aspiration, foreign body, edema). Treating the cause of hypoxia/hypoxemia must be done quickly because this is one of the potentially reversible causes of cardiac arrest [79]. Proper oxygenation and ventilation are key to restoring adequate amounts of oxygen into the system and negating lethal cardiac rhythm [80]. It is important to note that accidental aspiration of an element into the airways is a widespread clinical scenario among children under 3 years old, and it represents the leading cause of infantile death [81].

### 2.3. Central Nervous System

Among the nc-SDs, an important category is the imbalance of the autonomic nervous system control of the cardiovascular system. For these reasons, several neurological conditions, such as stroke, epileptic attacks and brain trauma, drugs, and catecholamine toxicity, may be related to SD [82].

Stroke represents the first cause of SD in this category; it is possible to distinguish three kinds of stroke:

- Intracerebral hemorrhage (ICH) secondary to hypertension or other causes. ICH represents about 10% of strokes. Hypertension is the most important risk factor in order to determine the risk for ICH: for example, Roberts et al. described two cases who died from non-traumatic ICH [83]. Moreover, in a recent study, Verdecchia et al. remarked its value as an independent prognostic marker for SD in the long-term [84].
- Brain infarction secondary to atherosclerosis or embolism. Moreover, a strong positive relationship exists between decreased heart rate variability (HRV) and SD [85]. Brain infarction is implicated in causing diminished HRV and is strictly associated with symptomatic carotid disease [86].
- Subarachnoid hemorrhage (SAH), secondary to ruptured berry aneurysm or other causes. It represents about 5% of stroke, and smoking, high blood pressure, increasing age, and possibly female sex are independent risk factors for SAH [87].

Other than stroke causes include:

- Bacterial meningitis: despite advances in clinical care, it remains a severe disease with a high risk of complications that may lead to SD [88];
- Epilepsy: it is named sudden unexpected death in epilepsy (SUDEP), referring to the sudden and unexpected death of an epileptic patient with no other health issues during normal activity. The exact cause of SUDEP has not been established yet; however, it is assumed to be caused by multiple organ failure [89];
- Brain tumor: even if it represents a rare event, an undiagnosed primary brain tumor may be considered a risk factor for SD. For example, Riezzo et al. described three cases of SD due to glioblastoma [90].

### 2.4. Abdominal Causes

The abdominal region could be involved in the generation of SD. Indeed, even if these causes are a less common cause of SD when compared to other conditions, they are equally important [91,92]. Particularly, the prevalent cause of death involving the abdominal region is massive bleeding into the peritoneal cavity or gastrointestinal tract: it could be linked to different diseases such as duodenal ulcer, gastric ulcer, ulcerative colitis or diverticulitis, malignancy, ruptured ectopic pregnancy, and ruptured viscus for the presence of bowels (e.g., ovarian cysts) [93,94,95].

Moreover, nc-SD involving the abdominal region could be generated by other diseases such as acute liver failure [96] or acute pancreatitis [97].

### 2.5. Endocrine System

Even if rare, endocrine diseases should be closely related to nc-SD. Notably, when other causes are excluded, this tragic event may be related to different pathological statuses such as adrenal insufficiency (although infrequent, it may occur in individuals treated for other critical conditions where impairment of corticoadrenal function often happen) [98,99], diabetic coma [100], severe hypothyroidism (myxedema) [101], parathyroid crisis [102,103], thymoma [104], etc.

### 2.6. Iatrogenic

The cases of nc-SD could be related to iatrogenic causes. In particular, even if it is frequently under-evaluated, it could be generated by problems related to prescription drugs [105]. Other causes may be related to the sudden withdrawal of steroids or other drugs. SD represents a complication of other tragic events related to medical errors, such as anesthesia or mismatched blood transfusion [106].

### 2.7. Miscellaneous

The so-defined miscellaneous category includes drug abuse: it could be related both to the assumption of controlled or uncontrolled substances [107]. In the first case, it is related to the assumption of legal drugs but with errors in the assumption (principally in older people) or voluntary wrong assumption (self-poisoning or for doping purposes), i.e., the use/abuse of anabolic-androgenic steroids (AAS). To date, AASs are frequently used not only to treat both hormonal diseases and other pathologies characterized by muscle loss, but by young people (athletes or individuals) to improve both physical appearance and performance [108,109]. AAS use/abuse is strictly related to the improved risk for SD [110,111].

In the other case, it is related to the assumption of drugs for “recreational” purposes [112]. For example, cocaine in its various forms could be closely related to fatal cardiac arrhythmia, microvascular injury, and acute myocardial ischemia due to coronary vasospasm are the most important causes of cocaine-related SDs. The cardiotoxicity of cocaine is not limited to massive doses of the drug, and underlying heart disease is not a prerequisite for cocaine-related cardiac deaths. Moreover, cocaine is associated with many health complications, including gastrointestinal ischemia/infarction and hemorrhage. For this reason, its assumption may generate SD [113]. Other recreational drugs such as heroin [114], marijuana [115], potent synthetic cannabinoids [116], as well as psychotropic drugs consumed by young people [117] may be related to SDs. Moreover, the proportion of users of recreational drugs was unexpectedly high, even more prevalent than other cardiovascular risk factors. Toxic effects could play an important role as triggers of SD, particularly in young people.

Other miscellaneous nc-SDs could be generated by anaphylaxis or bacteremic shocks, shock from dread, fright or emotion (vagal inhibition), sickle cell crisis, alcoholism, etc. [118,119].

### 2.8. Indeterminate

This category is reserved for those cases in which the cause of death remains in doubt even after an exhaustive study. Notably, to date, although the progress in the diagnostic fields, several deaths have been classified with the term ‘unascertained’: this should be reserved for circumstances where the cause and manner of death remain undetermined after autopsy [120].

## 3. Discussion

The mission of the forensic pathologist is to establish the exact cause of death. As previously described, in the case of SD, it represents a very complex task, although the scientific community has published professional guidelines, book chapters, and many scientific publications on this issue.

**Figure 2:**
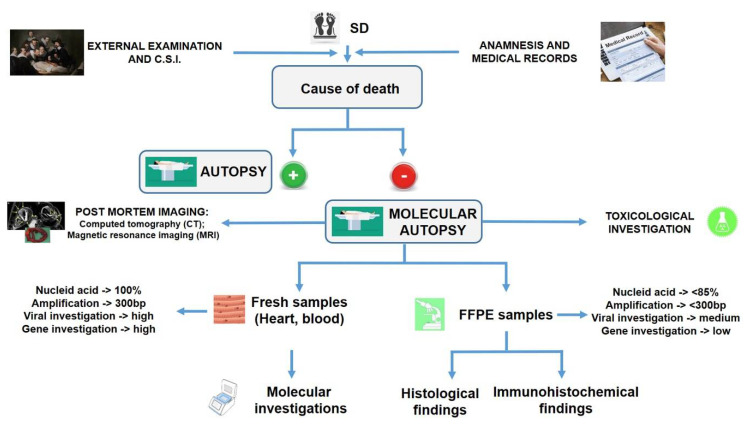
A practical flowchart that should be applied in all cases of SD.

In the definition of SD, it is important to clarify the meaning of the term “unexplained”: indeed, in common forensic practice, a “death” is defined “unexplained” only after an adequate post-mortem investigation. Obviously, from a forensic point of view, autopsy findings should be carefully evaluated as well as the crime scene investigation with the relative circumstances and the medical history of the victim. Even if the cause of death could remain unanswered after a thorough forensic investigation, from the medical standpoint it represents a critical situation with dangerous clinical implications. Particularly, SD potentially leaves family members at risk. In this scenario, the scientific community should improve efforts to define good practices in the case of SD. In this way, we suggest a practical forensic workflow that should be applied in every case of SD (Figure 2).

When an SD occurs, several steps should be conducted by the forensic pathologist in order to ascertain the exact cause of death. Obviously, the first two steps that should be performed with greater attention are the external examination and the crime scene investigation (C.S.I.). In the same manner, it is important to collect all information about the anamnesis and/or medical records of the victim. In this regard, several important data should be collected, such as age, gender, occupation, and lifestyle of the victim; the circumstances of death, past medical history, the presence of previous cardiovascular interventions, possible prescription of therapeutical drugs, family cardiac history, possible data collected during the rescue intervention (ECG tracking, serological data, etc.). In all cases, an autopsy should be considered mandatory in order to ascertain the exact cause of death. Before autopsy, the pathologist should collect numerous data from the external examination of the body: it is important to establish body dimensions (weight and height), checking for any dysmorphic features, skin, hair, or skeletal abnormalities, including the presence of a pacemaker or other external interventions (recent intravenous access, intubation, defibrillation, etc.). It is mandatory that all external and internal injuries should be described and photographed. Based on literature data [9], the forensic pathologist is able to directly determine a conclusive cause of death after macroscopic evaluation (positive macroscopic autopsy) in about 65% of cases (green signal), while it remains undefined in the other cases with the so-called “inconclusive autopsy” (negative macroscopic autopsy), indicated with the red signal in the flowchart.

In the cases with a negative macroscopic autopsy, further investigations are needed, applying the so-called molecular autopsy to explain the cause of death. As summarized in this graph, in cases of SD, it should be considered mandatory to perform the so-called “molecular autopsy”, meaning the application of molecular techniques to the post-mortem investigation. As summarized in the workflow, the samples collected during a standard autopsy protocol (fresh and/or fixed samples, for example, to perform toxicological and Microscopic Analysis) are used applying molecular techniques to identify hereditary diseases.In this context, it is important to stress the role of post-mortem imaging: several recent publications have reported the pivotal roles of computed tomography (CT) and magnetic resonance imaging (MRI) in order to identify different organ damage before the autopsy [121,122,123]. The usefulness of traditional X-ray images and post-mortem computed tomography (PMCT) is useful in visualizing calcified plaques, hemopericardium, and valves, and in identifying and locating cardiovascular devices [124,125]. Moreover, by means of a radiological investigation, different heart abnormalities can be highlighted [126]. As recently reported, modern radiological methods, such as multiple detector computed tomography (MDCT), MDCT-angiography, and cardiac MRI have been introduced into post-mortem practice for the investigation of SD, including cases of SCD [127]. A recent retrospective study studied the role of forensic post-mortem CT in order to define the cause of death, especially in cases of acute heart insufficiency or respiratory failure [128].

In the same way, a toxicological investigation plays a pivotal role in excluding the presence of exogenous substances that could be strictly related to SDs. Clinical and forensic toxicology represent two disciplines involving the quantification of xenobiotics in different biological and non-biological samples in order to define the diagnosis, treatment, prognosis and prevention of poisonings and to identify causes or contributory causes of death in cases of fatal intoxications [129]. The samples that could be collected to perform the toxicological investigation are: peripheral venous blood, vitreous humor, hair, urine, bile, pericardial/cerebrospinal fluids, and gastric contents. In a recent cohort study, Bjune et al. [130] reported that SCD victims with positive post-mortem toxicological findings showed polypharmacy assumption, showing this condition may play a proarrhythmic role in these cases. In 2019 Rippoll et al. reported that out of 101 enrolled SD cases, 52 showed positive toxicological findings. Ethanol was the most used substance, followed by legal drugs (meaning therapeutic drugs) and drugs of abuse. In general, the most used toxic substances are illegal drugs (especially cocaine), ethanol, tobacco, doping substances, and therapeutic drugs in not-recommended dosages [131]. Many prescribed drugs or illicit substances exert their adverse effects, both acute and chronic, on heart tissues: for these reasons, toxicological data are strictly related to the histopathological alterations of heart tissue [108,132,133,134,135].

As previously described, SD could represent the first manifestation of an unknown inherited cardiac disease. In similar cases, by applying genetic testing, it could be possible to discover the causality, with the identification of family member carriers, adopting preventive strategies [136,137]. Despite the fact that molecular autopsy is recommended in the guidelines for post-mortem investigation of SD, this is rarely performed, maybe because of the necessity to have a specialist laboratory [2,138]. Indeed, it is usually performed in cases of research projects, performing the analysis of the most prevalent genes associated with channelopathies (such as KCNQ1, KCNH2, SCN5A, and others), excluding other important candidate genes [18,139,140,141,142,143,144]. Moreover, in a recent article by Marey et al. [145], the pivotal role of post-mortem molecular testing in the strategy of family care after SD is remarked, particularly in suspected cardiomyopathy, since genetic findings provide additional useful information for relatives, which are beyond a conventional autopsy. In the last few years, the development of next-generation sequencing (NGS), which represents high-throughput genetic technology, has allowed the investigation of many candidate genes, reducing the bias of untested genomic regions [146]. The main problem related to NGS technologies is the necessity of ultra-specialist personnel and the costs. Indeed, to date, only a few studies have been performed proving the efficacy of this molecular approach in cases of SD [147,148,149]. In order to perform a molecular investigation, it should be mandatory to sample the victim’s body with different specimens, such as blood or other fresh tissues: indeed, the gold standard for molecular genetic testing is EDTA blood or fresh frozen tissue (heart, liver, and spleen) [150]. As summarized in Figure 2, formalin fixation and paraffin embedding (FFPE) of tissues are not recommended for molecular investigations, even if in the last few years technologies have improved the results obtained using these materials. Obviously, these samples are very important in order to perform histological and immunohistochemical investigations. These techniques are commonly used to highlight tissue damage; moreover, in SD cases, they should be used to identify different organ damage and/or the etiology in the myocarditis generated by different external agents, such as viral or bacterial infection: for example, the molecular techniques are the gold standard methods to diagnose viral myocarditis [151,152,153,154]. Identification of hereditary diseases is very important to extend the genetic analysis to members of the family of the deceased subject. An extensive, multigenerational family history offers the potential to improve every aspect of care: from establishing a diagnosis, to developing a genetic testing approach, to interpreting genetic test results, to continuously assessing the risk of SCD. Family history reconstruction could be considered not simply a static account of deaths and pre-existing diagnoses, but an ongoing dynamic process that incorporates new and valuable insights from family medical records, clinical cardiology assessments, genetic testing, and visual analysis of the deceased’s family tree [155].

In light of these data, following the suggested flowchart step-by-step, the forensic pathologist will be able to apply all the indications of the scientific community to real cases. Particularly, the data discussed in this narrative review suggest that autopsy should be considered mandatory in cases of SD. In this way, it will be possible to answer all questions relative to a SD: such as the death may be attributable to cardiac disease or to other causes, the nature of the cardiac disease (defining whether the mechanism was arrhythmic or mechanical), whether the condition causing SD may be inherited (with the subsequent genetic counseling), the assumption of toxic or illicit drugs, the presence of trauma, and other unnatural causes.

## 4. Conclusions

Based on this review, it should be stated that a Coroner’s post-mortem must be carried out in all cases in which SD occurs if the cause of death is unknown; obviously, in similar cases, the relative medical certificate of cause of death will not be forthcoming. Although forensic investigations may determine the cause of death in most cases, about 19% of cases remain unsolved, requiring further investigation. The molecular autopsy, thanks to modern technologies such as NGS, may help identify the cause of death in a large percentage of unsolved cases. The identification of new risk markers of SD remains one of the most important research fields for the scientific community [85]. For these reasons, a multidisciplinary working group of cardiologists, forensic experts, pathologists, intensive care specialists, geneticists, molecular biologists, and toxicologists is required [156]. Indeed, all clinical and forensic disciplines should build bridges between themselves [129]. Increasing relationships are improving the growth, reliability and the robustness of both kinds of laboratories.

In this context, it is interesting to report the experience of the Swiss Society of Legal Medicine, which in 2015 created a multidisciplinary working group composed of clinical and molecular geneticists together with cardiologists, in the hope of harmonizing the approach to the investigation of SCD [138]. This idea is strongly recommended in the guidelines for autopsy investigation of sudden cardiac death: the authors suggested their adoption throughout Europe with the aim of improving the standards of autopsy practice; moreover, they suggest the development of regional multidisciplinary networks of cardiologists, geneticists, and pathologists to collect more useful information [2]. Molecular medicine represents an important tool to improve the quality of death investigations, providing a new lens to better define the exact cause of death improving traditional methods [157]. Indeed, it is important to stress the key role that the medico-legal investigation has in SD investigations, not only in order to identify the exact cause of death but to indicate prevention in family members: high-resolution variant interpretation provides diagnostic accuracy and healthcare efficiency [158]. The autopsy report should conclude with a clear clinicopathological summary of the major positive findings and their relationship to the cause of death. Indeed, considering the importance of the genetic substrate, particularly in the case of SCD, the identification of mutations of lethal and inheritable cardiomyopathies and cardiac channelopathies can be applied in healthcare management.

Finally, it is important to remark that several causes of death remain unexplained after careful macroscopic, microscopic, and laboratory analyses: in this context, it should be considered mandatory to improve the research activity in this particular field, facilitating the identification of novel causes, and emerging patterns of diseases, causing SCD. In this way, each country should generate a multidisciplinary expert network to allow the interchange of knowledge in order to reduce “unexplained” deaths. Moreover, as recently suggested by Paratz et al. [159], another advisable action that could be adopted is the use of comprehensive multisource surveillance SCD registries that, even while they are not currently widespread, remain an appropriate method.

## Data Availability

Data sharing is not applicable to this article.
